# Cost-Efficient Oceanographic Instrument with Microfabricated Sensors for Measuring Conductivity, Temperature and Depth of Seawater

**DOI:** 10.3390/s24123940

**Published:** 2024-06-18

**Authors:** Matej Možek, Borut Pečar, Danilo Vrtačnik

**Affiliations:** Laboratory of Microsensor Structures and Electronics (LMSE), Faculty of Electrical Engineering, University of Ljubljana, Tržaška 25, 1000 Ljubljana, Slovenia; borut.pecar@fe.uni-lj.si (B.P.); danilo.vrtacnik@fe.uni-lj.si (D.V.)

**Keywords:** microfabrication, MEMS, conductivity, temperature, depth, NAUTILOS, marine measurement

## Abstract

The design, fabrication and characterization of a cost-efficient oceanographic instrument with microfabricated sensors for measuring conductivity, temperature and depth of seawater are presented. Conductivity and temperature sensors were fabricated using MEMS technology, which allows for customized small footprints and low production costs. Dedicated electronics for reading, processing and storing acquired sensor data are described. The developed instrument enables the measurement of seawater conductivity in a range from 4 mS/cm to 70 mS/cm. The conductivity measurement is temperature-compensated in the range from 2 °C to 40 °C, with an accuracy of ±0.1 mS/cm. The temperature sensor’s stability is 0.025 °C. The depth/pressure measurement range is up to 2000 m/200 bar, with a resolution of 0.1 bar. Temperature and conductivity sensor performance was assessed using laboratory equipment and designed electronics. The conductivity sensor was temperature-compensated to 0.01 mS/cm. The conductivity sensor electrode corrosion effect is presented below and was eliminated through adaptation of a signal acquisition circuit. Custom software was developed for monitoring critical conductivity sensor parameters (currents, voltages). A variation of 0.4% between cell conductance currents and voltages was established as a criterion for stable conductivity sensor operation.

## 1. Introduction

A CTD instrument is one of the most common instruments used by oceanographers, as differences in salinity, temperature and depth are the primary parameters, essential for understanding how the oceans affect the global climate. CTD is an acronym for conductivity (C), temperature (T) and depth (D). CTD will take a measure of conductivity, temperature and pressure (proportional to depth), and process these into a measure of salinity, thus monitoring seawater spatial and temporal variations. In general, an autonomous CTD instrument incorporates C, T and D sensors, electronic circuitry for distinct parameter setting, data acquisition, signal processing and storage, a power supply and connectors for remote control, all built in watertight pressure-resistant housing suitable for harsh seawater environments.

A CTD instrument can be used to profile a body of water in different ways. For example, a CTD instrument can be sent through a water column (surface to bottom or vice versa) to create a vertical profile of the water column. As the CTD instrument descends (or ascends), it records how the water changes, allowing researchers to see layers in the water column. In another mode of operation, a CTD instrument may be pulled through the water to identify horizontal variations (spatial variations). CTD instruments can also be fixed at a particular location and depth, allowing researchers to monitor water properties over time (temporal variations) [1]. CTD sensors are exposed directly to seawater. The chemical composition of seawater, along with its biota, introduces two main challenges in salinity measurements, as seawater is corrosive and rich in microorganisms that grow on solid surfaces and compromise sensor functioning [2]. Both pose a great challenge for long-term deployments, since the gathered data may be compromised by the latter.

All three variables are essential to determine salinity, which is the amount of dissolved salts in water. Salinity is usually determined as a ratio of the measured conductivity to the conductivity of a specific known concentration of dissolved ions. Salinity is formally expressed as parts per thousand, i.e., grams of dissolved salts per kilogram of seawater, but PSU (practical salinity unit) is considered more accurate as it considers more variables. Salinity is calculated using the TEOS-10 equations for practical salinity with data from the conductivity, temperature and depth (CTD) family of instruments [3,4]. Salinity, expressed in terms of the Practical Salinity Scale (PSS), is a dimensionless value, although by convention, it is reported as practical salinity units.

Although highly accurate CTD instruments are available from commercial companies, they are typically large and expensive. The development of low-cost instrumentation plays a key role in marine environmental studies and represents one of the most innovative aspects of current oceanographic research [5]. Recent developments of CTD instruments with a small size and low power consumption, suitable for mounting on gliders or AUVs and attaching to marine animals, have been reported in [6,7,8,9,10,11,12,13,14,15,16,17]. For near-shore oceanographic research on the continental shelf, where the water depth rarely exceeds 200 m, the cost expense of CTD research is a barrier for formal and informal researchers working with limited budgets, including scientists from developing nations, citizen oceanographers, environmental educators, and students of all levels interested in understanding their local coasts and waterways. One campaign oriented in that direction is Open CTD. Open CTD is a low-cost, open-source CTD instrument designed intentionally for the above-mentioned groups and was developed by a core team of marine ecologists in collaboration with a distributed community of scientists, engineers, makers and conservation practitioners [18]. One of the goals of the presented CTD instrument is to extend the depth limit of 200 m, set by the Open CTD campaign, to 2000 m, which is a demanding environment from several aspects. Despite this, the low-cost aspect of the presented CTD design was a principle criterion during our development stage. Commercial CTD probes use typically large and expensive conductivity cells.

Miniaturized CTD probes, such as the presented case, are using smaller cells, based on microelectromechanical system (MEMS) technology [13,14,15,16,17,18]. The MEMS approach represents an alternative to conventional conductivity cell design, although perhaps at the expense of accuracy. On the other hand, it offers the ability to be mounted on even smaller platforms [8], combined with other devices. Besides, it has lower power consumption, i.e., achieves a longer deployment time.

Microelectromechanical system (MEMS)-based (CTD) sensors offer miniaturization, which is useful for distributed networks with a large and dynamic sampling volume and for adequate accuracy of measurement. The advantages of using MEMS-based sensors are as follows: (1) negligible creep and fatigue from hysteresis, (2) the capability of integration and batch fabrication and (3) simple interfacing to electronic circuits. Furthermore, MEMS thin-film sensors are characterized by their lightweight nature, flexibility, low power consumption and low fabrication costs. In addition, redundancy can be achieved using miniaturized sensors, since several CTD setups can be accommodated in an instrument with marginal mass and volume penalty. Another expense associated with commercial instruments is the maintenance and recalibration, which is typically performed by the manufacturer. This contributes to instrument downtime, whereas the MEMS sensors can be expendable and replaced due to their cost-effectiveness, minimizing the downtime of the entire system. Based on the above facts, we adopted MEMS thin-film technology as our leading approach in the development of a cost-efficient CTD instrument with a miniature, microfabricated CT sensor suitable for deep-ocean water monitoring to a depth of 2000 m.

In the following, self-contained microprocessor-controlled highly accurate electronics for measuring the C, T and D of seawater will be presented.

## 2. Instrument Description

**Figure 1:**
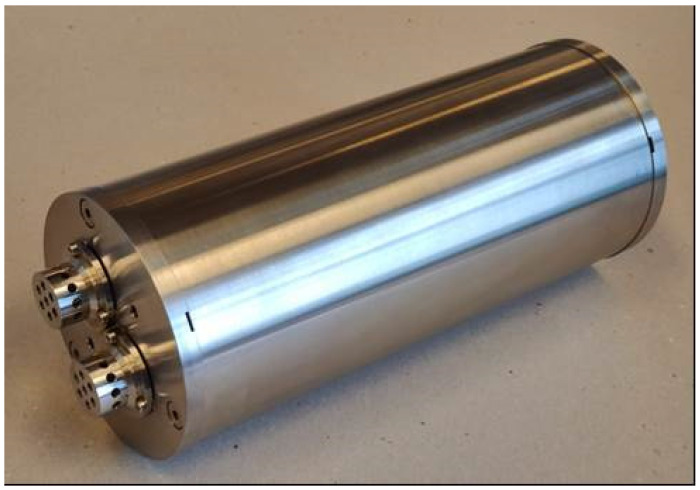
CTD instrument built for deep-ocean deployment. Housing made from SS 316L, with length of 300 mm and diameter of 120 mm.

The presented CTD instrument comprises an integrated MEMS-based microstructured thin-film electrode (mTFE) conductivity cell and RTD temperature sensor with a separate OEM Keller pressure sensor and electronic circuitry for control and signal processing, all assembled in watertight pressure-resistant housing suitable for seawater measurements to a depth of 2000 m (Figure 1). All three sensors were packaged in two openings in the top cover of the CTD housing made of stainless steel material (SS 316L), considered the most corrosion resistant.

Connectors and cables are attached to the connection pads of the conductivity and temperature sensors. Sensors are potted by epoxy glue in an SS case, fitted to one of two watertight ports of the metal housing. The housing front cover plate (Figure 1) comprises CT sensors which are mounted in the upper port, while the pressure sensor is mounted into the lower port, with connecting wires extending downwards to the PCB inside the SS cylindrical body. All sensors, electrical connectors and front and back cover plates are sealed with Viton^®^ O-rings on the cylindrical body. The watertight SS 316L cylindrical housing accommodates the PCB of the CTD electronic circuitry and all necessary connections to the sensors and outside connector. The electronic PCB is placed inside the cylindrical housing, where it is fastened to the front cover of the CTD housing. The back cover plate contains an underwater connector for communications and power supply. Data are recorded in the instrument memory and are simultaneously available at RS-485 I/O watertight connector pins. The whole system has a diameter of 120 mm and a length of 300 mm, intended to accommodate autonomous power supply in future instrument extensions. The achieved CTD instrument technical characteristics are listed in Table 1, which lists the main properties such as measurement range, accuracy and resolution for conductivity, temperature and depth sensors. A typical ocean seawater conductivity range is up to 50 mS/cm, while the presented CTD instrument’s measurement range is from 4 to 70 mS/cm, with an accuracy of ±0.1 mS/cm and a resolution of 0.01 mS/cm. The sensors exhibit linearity of (R_sqr_ = 0.998) over the entire measurement range. It offers temperature compensation in the temperature range (0 to 40 °C). The implemented titanium temperature sensor achieves a sensitivity of 4450 ppm/°C, which is better compared to its platinum counterpart (Pt1000 3850 ppm/°C), which is typically used in comparable CTD instruments. Detailed analysis of the summarized parameters is shown in the Results Section.

**Figure 2:**
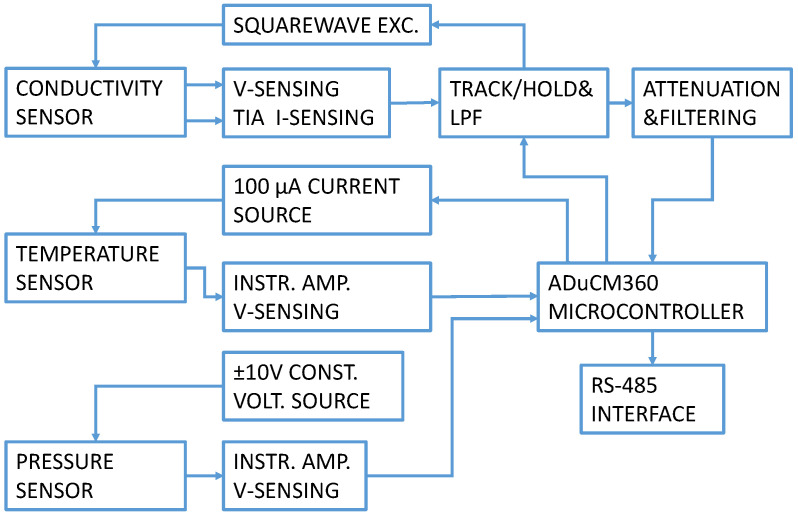
CTD electronics circuit block diagram.

A CTD electronics block diagram is presented in Figure 2. The electronic circuit generates a precise AC square wave excitation voltage with minimum DC offset to avoid damaging the conductivity sensor electrodes. The excitation amplitude and frequency are user-programmable. An innovative synchronous sampling technique converts the peak-to-peak amplitude of the excitation voltage and current to a DC value for accuracy and ease in processing using the dual 24-bit Σ−Δ ADC contained within the precision analog microcontroller. In our CTD instrument’s electronics, conductivity cell and temperature sensor signal conditioning were based on the Analog Devices CN0359 development kit [19] and adapted to our sensor properties. A pressure/depth sensor measurement channel was added to the existing development design with a separate constant current excitation, signal conditioning and temperature compensation.

Control and setup are implemented using the RS-485 interface. The circuit operates from a single power supply between 13.5 V and 16.5 V. Current consumption in continuous measurement mode with all sensors connected and connection established is 50 mA.

### 2.1. Conductivity Sensor

There are two main types of conductivity sensors in use for oceanographic studies: electrode and toroidal or inductive sensors. Inductive sensors are relatively large and bulky due to the toroidal case. Electrode sensors contain two, three, four or seven electrodes [10]. Conductivity is a measure of the water’s ability to conduct an electrical current and is measured in Siemens per meter (S/m). Regarding seawater, the higher the electrical conductance, the more dissolved salts are present in the given sample and therefore the higher the salinity. Changes in the conductivity could indicate a change in water mass or an influx of salts or other minerals, possibly caused by seasonal changes, pollution or other parameters. Variables affecting conductivity are temperature, pressure and dissolved inorganic compounds. The amount of dissolved solids (salts as a major factor) in a sample of water is a dominant variable in conductivity measurements but there is also a pronounced temperature dependency and a minor pressure dependency. The conductivity of water will increase as the temperature increases. A 1 °C increase in the temperature of seawater will typically result in a 1.8–2.1% increase in conductivity [20], expressed as the temperature coefficient of conductivity (TCC) in the following. This is due to an increase in ionic mobility as well as the increased solubility of many salts and minerals. Because of this dependency, water conductivity is measured as specific conductivity with measurements performed at (or corrected to) a standardized temperature, usually 25 °C. The following equation summarizes how conductivity compensation is obtained from conductivity measurements:

(1) $$C_{25}=\frac{C\left(T\right)\cdot 100}{100+{TC}_{C}\cdot \left(T-25\right)}\;C\left(T\right)=\frac{k_{C}}{R_{C}},$$

where *C*_25_ is the conductivity at a standardized temperature and *TC_C_* is the temperature coefficient of conductivity. Measured conductivity at an ambient temperature *C*(*T*) can be further expressed as a ratio between the proportionality coefficient (cell constant, *k_C_*) and temperature-dependent resistance (*R_C_*) or a product with conductance (1/*R_C_*). The cell constant depends on the geometry of the electrodes and the geometry and size of the water volume in the vicinity of the electrodes. The cell constant *k_C_* will be modified if any insulating or conducting object moves closely into the electric field; this is called the proximity effect. To confine all of the electric field close to the sensor area, open conductivity cells may be manufactured in the form of microelectrodes, as presented here. These designs do not require a large volume of surrounding media.

In comparison to standard professional inductive or large circular multi-electrode conductivity cells, which are most prevalent in oceanographic instruments, this paper focuses on planar, miniature thin-film microstructured gold electrodes. This approach was implemented due to the following advantages: sensor miniaturization, easy sensor replacement, lower sensor manufacturing cost using batch fabrication, low power consumption of the sensor signal-conditioning circuit and the ability of conductivity cell integration with an RTD temperature sensor.

On the other hand, cost efficiency has an impact on accuracy and/or lifetime, which are expected to be lower compared to the high-accuracy professional devices on the market. However, since our focus is predominantly on a large number of sensors at a reduced cost, covering spatial and temporal gathering of data, this is believed to be a viable approach.

Within this study, a four-electrode conductivity sensor is designed to reduce the electrode polarization effect, which is usually a drawback observed for a two-electrode configuration.

**Figure 3:**
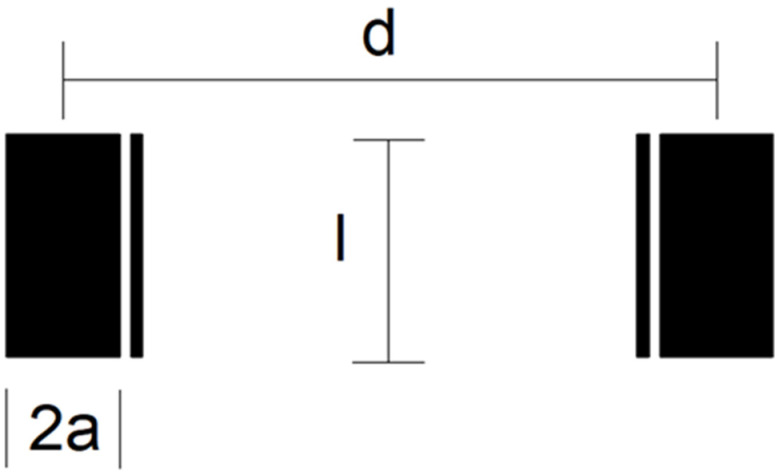
Schematic diagram of electrode design geometry of conductivity sensor.

Three different electrode design geometries (C1, C2 and C3) with chip dimensions 14 × 8 × 0.6 mm^3^ were fabricated. The four-electrode conductivity cell’s geometric detail is depicted in Figure 3. The outer (wider) electrodes are current electrodes, while the inner (thinner) electrodes are voltage electrodes. Electrodes differ in their area (A), length (l) and inter-electrode distance (d). The width of current electrodes is set with parameter 2a, while the width of voltage electrodes is fixed (100 µm). The distance between each current and voltage electrode is also 100 µm.

Electrode dimensions with the measured cell constant *k_C_* are listed in Table 2, while the constant is determined based on the measurement described in Section 4.1. We found that higher electrode spacing lowers the constant *k_C_* by around 10% and, consequently, the sensitivity of the conductivity sensor. The cell constant can be specified at any value within the measurement range of salinity due to the linear correlation between conductivity and salinity of water.

### 2.2. Temperature Sensor

A dedicated temperature sensor is used for the measurement of water temperature variations (spatial and temporal profiles) as an independent variable. The same temperature readouts are also applied into a correction algorithm of conductivity. Finally, the measured values are also used for temperature compensation of the measured pressure, where the temperature dependence of the piezoresistive effect requires temperature compensation as well.

The most frequently used sensor for measuring the temperature of seawater is an RTD (Resistance Temperature Detector) or a thermistor. RTDs use a sensing element whose resistance varies with temperature, usually with a positive temperature coefficient. Different metals will resist an imposed electric current differently at different temperatures, so the change in the measured voltage drop will reflect the temperature.

A constant current is passed through metal strip electrodes, the voltage drop is measured and the resistance is calculated. Care has to be taken to apply a low electrical current for sensor excitation to avoid joule heating (self-heating), which affects the accuracy of measurement. Using conversion coefficients, this change can be used to determine the temperature of the water with an extremely high degree of accuracy.

Thin-film temperature sensors demonstrate linear behavior in a wide temperature range, have high sensitivity and represent a small mass. They are desirable particularly where low power consumption and fast thermal response are essential. The electrical properties of thin films such as resistivity and the temperature coefficient of resistance (*TC_R_*) depend on the microstructural properties and surface morphology. Surface morphology is further strongly related to the deposition process, thermal history and material purity. Electrical resistance of the thin-film metal sensor is expressed as a function of temperature:

(2) $$R_{T}=R_{0}\cdot \left(1+{TC}_{R}\cdot \left(T-T_{0}\right)\right),$$

where *R_T_* is the sensor resistance at temperature (T), *R*_0_ is the sensor resistance at the reference temperature (*T*_0_) and *TC_R_* is the temperature coefficient of resistance at the reference temperature. Our calculations take into account *T*_0_ = 0 °C to comply with the DIN/IEC 60751 (IEC751) standard [21].

A prevalent thin-film RTD sensor fabrication material is platinum. However, platinum is an expensive material, is more difficult to process and requires an additional prime adhesive layer (Cr or Ti). Due to its lower sheet resistivity, it is difficult to obtain high resistance values on a small area. Finally, it also exhibits lower sensitivity (*TC_R_*) than, e.g., nickel or titanium.

Instead of platinum, the material of choice for the fabrication of an RTD in this study is a titanium thin-film resistor fabricated using thin-film MEMS technology and integrated on the common substrate with the conductivity sensor. A thin-film titanium temperature sensor is made on a low-thermal-conductivity glass material (Borofloat 33, heat conductance 1.2 W/mK) which thermally isolates the Ti sensing meander resistor from the massive SS316L (16.3 W/mK @ *R_T_*) case. Additionally, a glass substrate was chosen to minimize unwanted parasitic capacitances, which are important in conductivity measurements [9].

**Figure 4:**
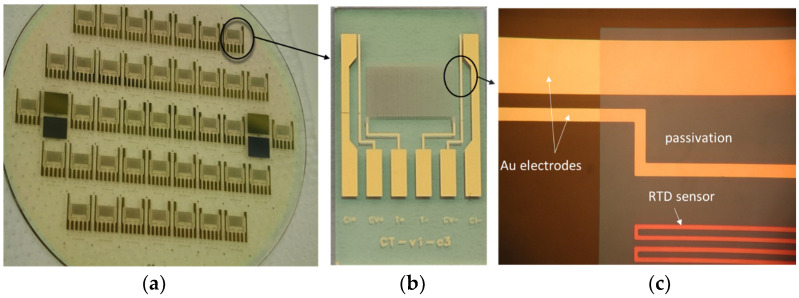
Details of fabricated CT sensor: glass wafer with CT sensors (**a**), single CT sensor die (**b**) and details of C and T sensor (**c**).

The titanium meander-like resistor geometry is 168 mm long, 40 μm wide and 780 nm thick, resulting in a resistance *R*_0_ of 2950 Ω and a *TC_R_* of 4450 ppm/°C. The design of the Ti RTD sensor should yield as high a meander resistance as physically possible (note—RTD width is limited by conductivity sensor inter-electrode distance; see Figure 4b). This would enable the use of lower DC bias currents with a higher RTD voltage response. Lower DC bias currents mitigate potential error due to the self-heating effect, while higher *R*_0_ values minimize the impact of electrical connection serial resistance. The titanium resistor is positioned in between the voltage electrodes of the conductivity cell and protected by a thin-film silicon nitride layer (1.2 W/mK @ *R_T_*). Passivation using high-quality SiN retains a stable and reliable long-term seawater operation, because SiN is insensitive to water uptake. Sensor integration provides temperature measurements at a minimal distance from the conductivity measurement sampling point.

The advantages of the proposed thin-film Ti RTD over a thin-film Pt RTD are numerous: a reduction in material cost (Ti vs. Pt), the simplification of fabrication (etching vs. lift off, single layer deposition), more accurate line width transfer and better repeatability, a higher *R*_0_ for the same dimension (specific electrical resistivity is approximately four times higher (42 µΩcm with respect to 10.6 µΩcm of Pt)), a higher *TC_R_* (4450 ppm/°C as compared to 3850 ppm/°C for Pt, i.e., a 17% increase) and fast response time.

### 2.3. OEM Depth Sensor

Depth is a less significant parameter for correct salinity measurement, but still an important parameter for supporting other environmental ocean variables. The depth/pressure sensors in stationary CTD instruments are so sensitive that they can accurately measure the tidal cycles—the alternating high and low tides every 24 h—or even waves’ alternating crests and troughs on a time scale of seconds.

In general, pressure sensor design is based on a flexible membrane. Deflection of the membrane, induced by the applied pressure, is converted into an electrical output through a component that is sensitive to diaphragm deflection (capacitive coupling) or associated stresses (piezoresistors, strain gauges). Most commonly, commercially available pressure sensors are based on a piezoresistive principle and also include temperature compensation. Industry standard silicon pressure sensors with fully thermally compensated circuitry are generally used as stand-alone sensors, while integration with CT on the same substrate is less common.

Besides modularity and maintenance, the replacement cost of integrated sensors is one of the reasons to separate pressure sensors from CT sensors.

In this study, the pressure transducers from the Keller PA-4L series were chosen because of their small and compact design, making them ideally suited to OEM applications with limited installation space [22]. The pressure sensor is an absolute pressure sensor with piezoresistive readouts, optimized for a full pressure range of 200 bar. The piezoresistors are placed in a Wheatstone configuration on a square membrane, protected by an SS membrane welded on the front side, with a low-compression medium in between the two. The metal diaphragm is in direct contact with seawater. Each pressure transducer is measured over the entire specified pressure range and their accuracy is determined as the percentage of the full scale of the pressure range. For each pressure sensor, a dedicated table with the resistance values of the compensation circuit is given, which ensures the sensor performance in a wide temperature range (−20 to +85 °C).

### 2.4. Thin-Film MEMS Fabrication of CT Sensors

Conventional thin-film MEMS technology was used in the fabrication process. The substrate material was Borofloat 33 glass from Schott (New York, NY, USA), 100 mm in diameter and 700 µm thick. First, a prime layer of Cr (30 nm), followed by a layer of Au (200 nm), was deposited by using the sputtering method on the substrate. The designed and elaborated photomask was used to transfer and define the shape and geometry of electrodes by following standard photolithographic steps. This was followed by a wet etching process to remove unprotected Au and Cr, thus leaving behind well-defined Cr/Au electrodes and connecting pads. Since the temperature sensor is located on the same chip, the processing continued with the deposition of the Ti layer (750 nm) performed as well by using the sputtering deposition method. The following photolithographic step defines the shape and size of the meander-like Ti resistor, representing an RTD temperature sensor. After dry etching of the exposed Ti regions and removing the photoresist, final C and T sensor structures are revealed.

In order to confine the region of the conductivity electrode area that will be exposed to the medium and to protect the Ti meander, an insulating passivation layer was applied. This was achieved by depositing a 1200 nm thick PECVD silicon nitride layer, which is the final step after defining the essential regions (Au electrodes and contact pad remain unprotected), removed by dry etching. Figure 4 shows the fabricated CT sensors from a wafer to a chip.

**Figure 5:**
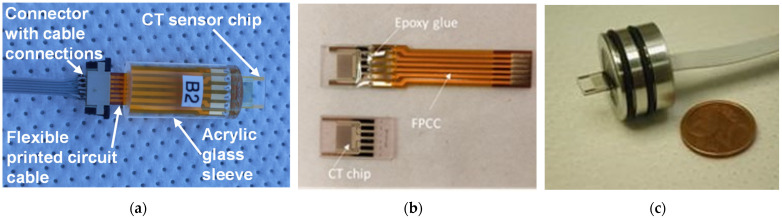
CT sensor encapsulated in different housings: (**a**) and (**b**) for laboratory measurements and characterization and (**c**) for deep-ocean deployment (2000 m).

The following step is dicing them into individual chips and connecting sensors via connection pads glued to a flexible printed circuit cable (FPCC) using conductive Ag glue (silver-filled epoxy EPO-TEK EE 129-4 (Paisley Products of Canada Inc., Scarborough, ON, Canada)). The final step is encapsulation into one of three housings, shown in Figure 5, through potting with epoxy glue EPO-TEK 302-3M [23].

**Figure 6:**
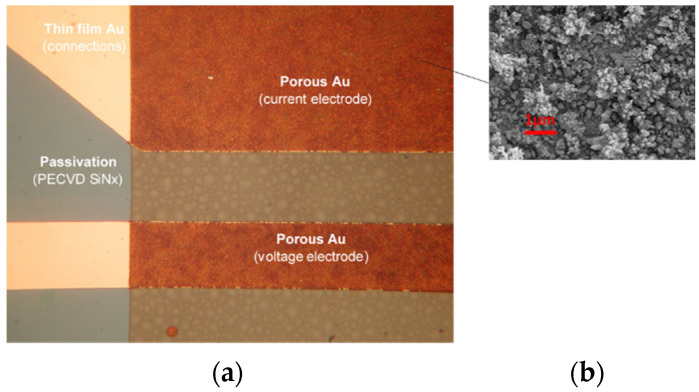
Microstructured electrode detail of conductivity sensor: (**a**) optical microscope and (**b**) SEM of microstructured electrode surface.

To overcome problems with capacitance, limited by a small electrode area, microstructuring of the thin-film gold electrode surface was applied. The obtained increased capacitance of the metal/electrolyte interface results in a less frequency-dependent, more stable and faster sensor response with respect to the thin-film gold electrodes. Microstructured gold electrodes were implemented on the finalized CT sensor die through selective electrochemical deposition in 3 mM HAuCl_4_ in 0.1 M NH_4_Cl, using the pulse amperometric deposition method. The result of Au electrochemical deposition was microstructured surfaces with thicknesses between 1 and 2 µm and a highly dendritic surface; see Figure 6b.

### 2.5. Design and Circuit Description of CTD Electronics

**Figure 7:**
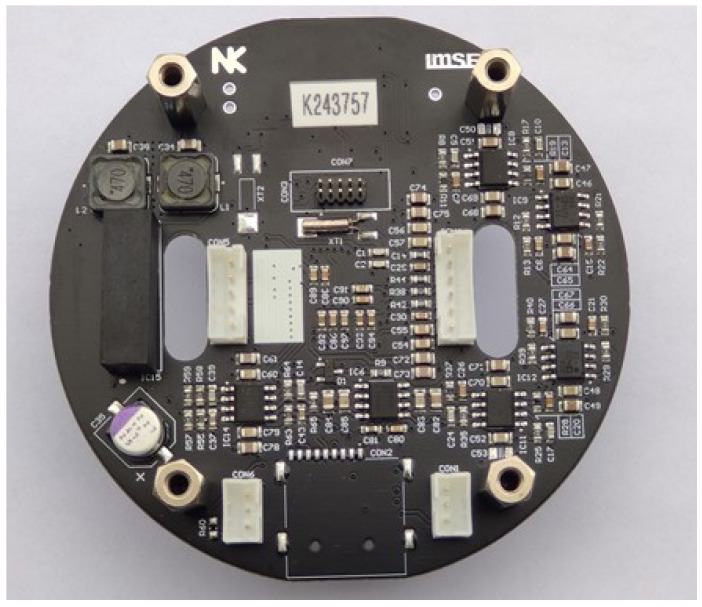
Four-layer PCB of complete CTD electronics.

The realized CTD electronics, presented in Figure 7 as a final unit, can be divided into separate conductivity, temperature and pressure sensor signal-conditioning circuits, each described in detail in following sections.

Analog power supply voltage (±15 V) is generated using Murata MEA1515S (IC1) (Murata Manufacturing, Nagaoka, Japan), a 1 W compact switching power supply module with improved regulation performance ≤5% and typically 5 mV_PP_ noise regulation (max. 20 mV), capable of supplying ±33 mA on each power supply rail +15 V and −15 V. Its input power supply voltage range is between 13.5 V and 16.5 V, making it ideal for battery-powered applications (e.g., 14.4 V Li-ion battery or similar).

Conductivity cell excitation is achieved using a square wave generator, with adjustable frequency and amplitude. It is imperative that the square wave has a precise 50% duty cycle and a very low DC offset, since even small DC offsets can destroy the cell due to electrode polarization over time.

**Figure 8:**
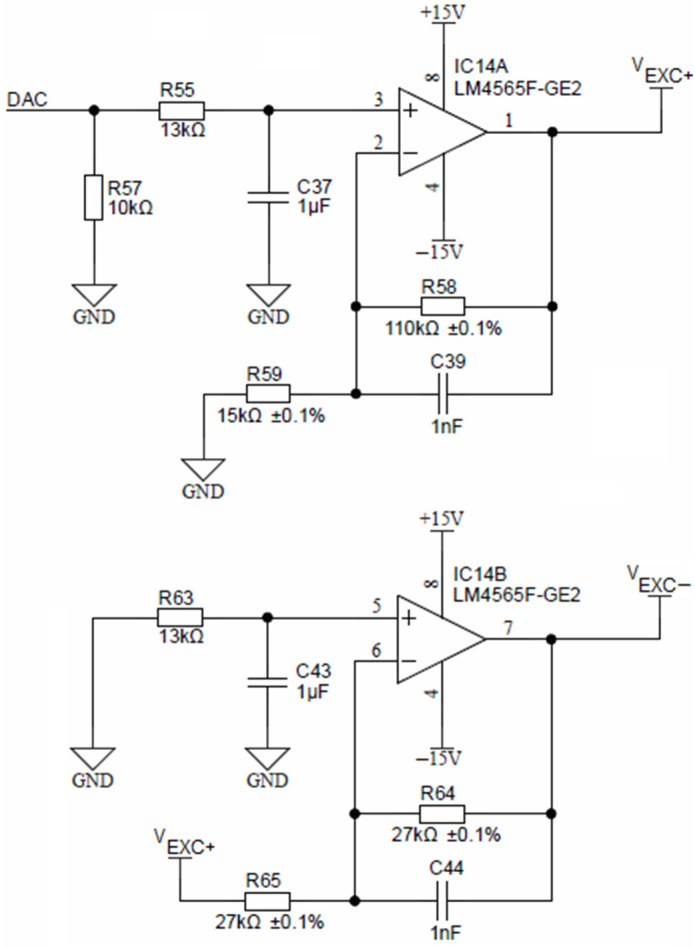
Excitation voltage sources.

Precise voltage symmetry is achieved using a non-inverting amplifier, comprised of a low-offset operational amplifier LM4565 and two resistors *R*_55_ and *R*_58_ (Figure 8). The non-inverting amplifier is fed by a DAC voltage output of the ADuCM360 microcontroller.

The non-inverting amplifier, shown in Figure 8, has a fixed gain of 3.33, which translates a 3.3 V DAC output to any analog *V_EXC+_* voltage up to 10 V in 12-bit resolution. Representation of the negative amplitude (*V_EXC−_*) is achieved by inverting the amplifier (again LM4565) with a constant gain of −1, formed by two equal resistors *R*_64_ and *R*_65_.

**Figure 9:**
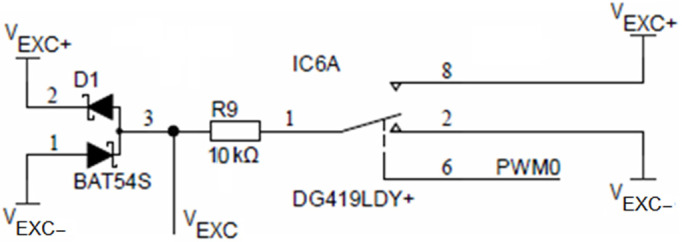
Conductivity sensor excitation.

Time symmetry of the conductivity cell excitation voltage *V_EXC_* is achieved using an SPDT analog switch DG419, which is alternating between *V_EXC+_* and *V_EXC−_* voltages using the PWM0 output of the ADuCM360 microcontroller, as depicted in Figure 9. The DG419 SPDT analog switch features 18 Ω on-resistance with an on-resistance flatness of 1 Ω over a ±15 V range. The voltage symmetry error introduced by the switch is typically 18 Ω/10 kΩ = 0.18%. Resistor R_9_ limits the maximum current through the sensor to 10 V/10 kΩ = 1 mA. Driving current selection is the primary limiting factor in the upper limit of the conductance measurement. The expected conductance is not to exceed values above 100 mS. Driving current reduction also attributes to lower overall current consumption. The two Schottky diodes (D_1_, BAT54S) form a clamper to +*V_EXC_* and −*V_EXC_* voltage extrema.

For the conductivity sensor, voltage and time symmetry (i.e., both temporal and voltage half-wave symmetry) are essential to minimize the polarization effects of electrodes and consequentially their electro-corrosion. Time symmetry of the conductivity cell excitation voltage V_EXC_ is achieved using an analog switch, which is alternating between V_EXC+_ and V_EXC−_ voltages using the PWM0 output of the ADuCM360 microcontroller, as depicted in Figure 9. Time symmetry deviation is defined by microcontroller oscillator stability (typ. 50 ppm). The voltage symmetry error introduced by the switch is typically 18 Ω/10 kΩ = 0.18%. Voltage symmetry deviation is defined by R_64_ and R_65_ resistor matching (inverting amplifier, IC14B, gain −1.0, see Figure 8) as well as on-resistance flatness of the analog switch (see Figure 9), which is 1 Ω over a ±15 V range. In order to protect the circuit from the short-circuit condition, resistor R_9_ was added. This resistor limits the maximum current through the conductivity sensor to 10 V/10 kΩ = 1 mA. At the same time, the AC voltage source V_EXC_ and R_9_ form a current source, which can be set to appropriate values to avoid excessive electrode driving current. Electrode driving current selection is the primary limiting factor in the upper limit of the conductance measurement. 

V_EXC_ is determined so that at higher salinity (0.8 M NaCl), the electrode voltages (V_PP+_ and V_PP−_) are to be minimal reliable detectible values (≥±10 mV). This results in V_EXC_ being higher than 1.2 V and in our experiments, it is applied at 1.6 V.

**Figure 10:**
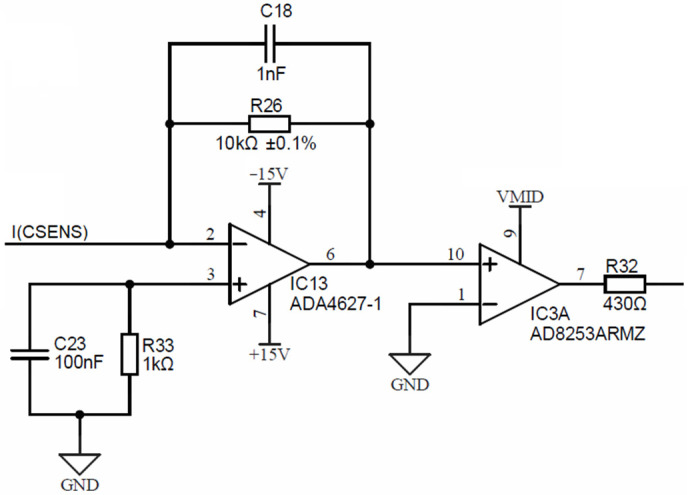
Conductivity cell current electrode input stage.

Figure 10 is depicting a conductivity cell current electrode input I(CSENS), which is input to the precision current-to-voltage converter, comprised of a low-bias current (1 pA), a low-offset (120 μV) operational amplifier IC13 (ADA4627) and a precision resistor R_26_, which translates the input current to voltage.

The symmetry error, produced by the 120 μV offset error, is only 12 ppm at a 10 V range. The output of the current-to-voltage converter is input into IC3, which is a software programmable gain amplifier (PGA) AD8253, with decade gain settings (1, 10, 100, 1000). This PGA will amplify the converted current readout and introduce a DC voltage shift to center point *V_MID_*, set at exactly half of the 3.3 V power supply voltage of the ADuCM360 microcontroller. The output of the PGA (resistor R_32_) is connected to the synchronous modulator stage, presented in the following.

**Figure 11:**
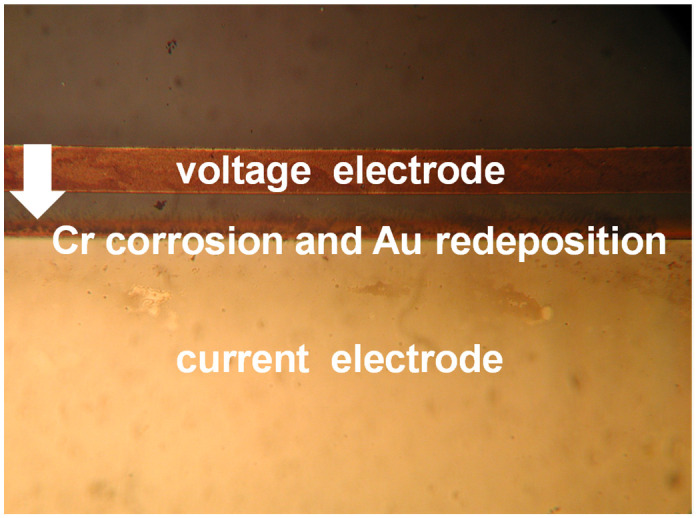
Gold redeposition from voltage to current conductivity sensor electrode.

In the preliminary stage of conductivity measurements, the original CN0359 kit design implemented a floating conductivity voltage electrode connection. Such configuration would cause gradual electrode corrosion and final conductivity sensor malfunction in several hours. Corrosion starts from redeposition of the metal film from the voltage electrode to the current electrode (Figure 11) and typically in few hours completely dissolves the voltage electrode.

**Figure 12:**
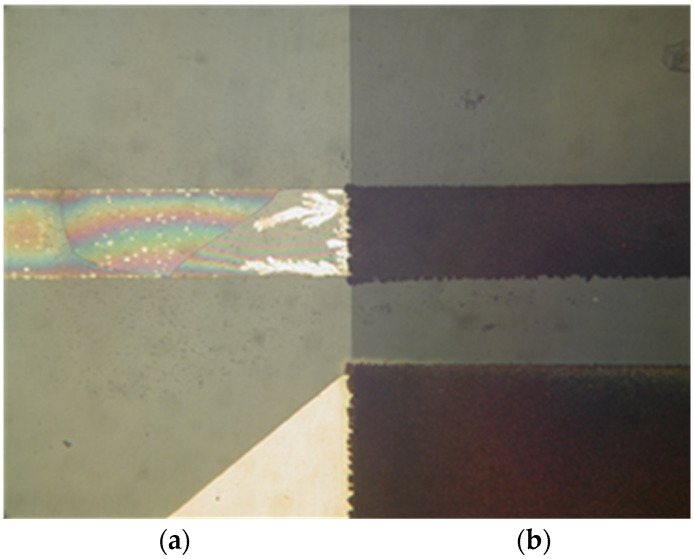
Electro-corrosion of exposed voltage electrode (**a**) and preserved area, protected by silicon nitride (**b**).

Tested sensors were inoperational after four hours. It was noted that Au voltage electrodes were substantially corroded and the chrome prime layer beneath Au was fully etched away (Figure 12). The protected area with the interface layer, covered by silicon nitride, was intact, and so was the RTD sensor. From this result, we could conclude that a substantial charge transfer occurred between the voltage and current electrodes of the conductivity sensor. This could be attributed to unexpected DC bias due to floating voltage electrode potential.

**Figure 13:**
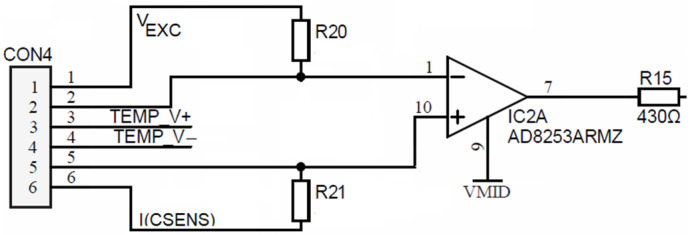
Conductivity cell’s voltage electrode large resistor biasing.

The described corrosion process was eliminated through fixation of voltage electrodes to a potential, which is lower than the redox potential that prevents the transfer of electrode material from voltage to current electrodes. Additional current flow, presumably caused by input bias currents of the programmable gain amplifier (PGA), elevates the individual voltage electrode potential over its redox potential value. This over-potential reduction was achieved with the insertion of two high-(order of MΩ) resistors between input terminals of the current and voltage electrodes, as depicted in Figure 13. The bypassing current through the resistors is in the range of a few hundred pA and does not distort the performance of the sensor, where typically, the injected electrode current is a few hundred microamperes. Any variation in individual voltage electrode potential by itself is cancelled out by the conductivity sensor acquisition principle, described in Section 2.5.1.

Voltage electrodes are connected directly (DC coupled) to inputs of IC2, which is a (PGA) AD8253. Each PGA input pin bias current is coupled to cell excitation using high-ohm (order of MΩ) resistors *R*_20_ and *R*_21_ in order to prevent the above-described corrosion effect. Such high-value resistors vaguely fix the potential of the corresponding current electrode and, at the same time, provide a current path for equalization between PGA input currents. Hence, such a high-resistance chain prevents the forming of a floating DC potential, which occurs due to charging SiN passivation (Figure 11), by flowing a small constant current through the PGA input stage.

**Figure 14:**
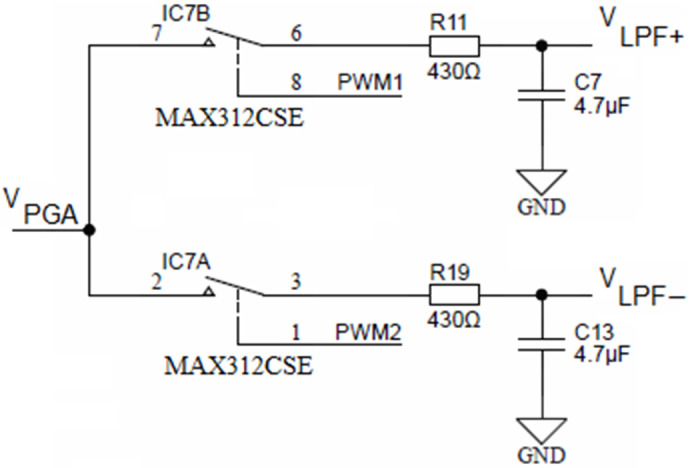
Synchronous sampling stage (simplified).

Both PGA outputs (i.e., R_32_ from Figure 10 and R_15_ from Figure 13) are connected to two separate synchronous modulator stages and each of these are comprised of two analog SPST switches MAX312CSE, each switch followed by its low-pass RC filter, as shown in Figure 14.

The upper SPST switch IC7B is controlled with the PWM1 output of the ADuCM360 microcontroller, while the lower switch (IC7B) is controlled with the PWM2 output. Each output of the low-pass filter with a cut-off frequency ca. 78 Hz is connected to an operational amplifier, configured as a buffer. The role of the synchronous modulator is to provide a track-and-hold function for each half-cycle of conductivity cell excitation voltage *V_EXC_*.

**Figure 15:**
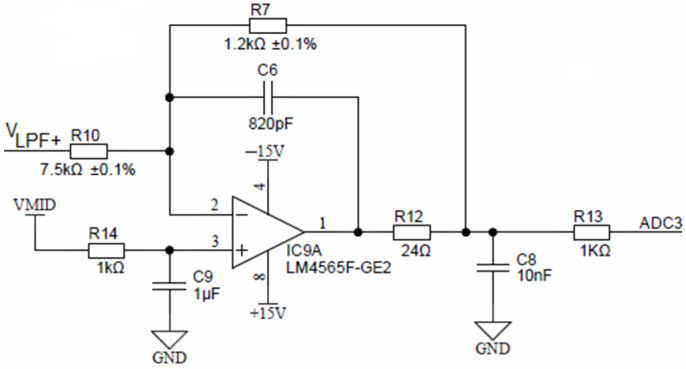
Final attenuation and filtering stage.

Buffered low-pass filter voltages (same as *V_LPF+_* and *V_LPF−_*) are connected to their corresponding inverting attenuator. Figure 15 shows one of four equal attenuators with IC9A for V_LPF+_ input. These attenuators each provide attenuation of −0.16 and a DC shift to a common-mode output voltage of *V_MID_* of 1.65 V. The attenuator stage reduces the maximum input signal of *V_EXC_* = ±10 V to ±1.6 V, with a common-mode voltage of +1.65 V, which is compatible with the ADuCM360 differential input ADC range. Attenuator stages also provide noise filtering and have a cut-off frequency of approximately 198 kHz.

#### 2.5.1. Conductivity Measurement Principle

**Figure 16:**
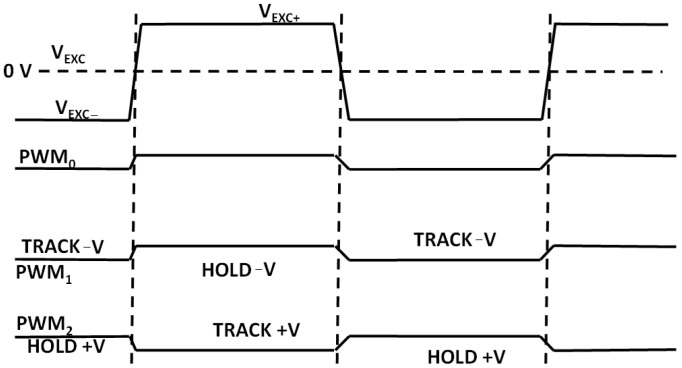
Simplified cell excitation voltage and sampling PWM signal polarities.

The microcontroller ADuCM360 generates the PWM0 square wave switching signal for the DG419 switch (Figure 9) as well as the PWM1 and PWM2 synchronizing signals for the synchronous sampling stages (Figure 14). The cell excitation voltage and PWM timing waveforms are shown in Figure 16. The process of obtaining the conductivity measurement result is divided into a positive and negative sampling stage—based on V_EXC_ polarity—when the sampling occurs.

Each stage is repeated for conductivity cell voltage and current electrode measurements. During the positive-V_EXC_ stage, sampling PWM1 is in-phase with excitation PWM0 and PWM2 is in counter-phase with PWM0. The resulting differential ADC readout for positive V_EXC_ can be expressed as *V_p_volt_*. Afterwards, the same sampling process is repeated for negative *V_EXC_* with the PWM1 inverted (i.e., counter-phase with PWM0). The track-and-hold phases in Figure 16 are reversed and the obtained cell voltage readout is *V_n_volt_*. The same process occurs for conductivity cell current readouts—resulting in ADC voltage readouts of *V_p_curt_* and *V_n_curt_*. The obtained voltages can be recalculated to measure the conductivity cell current I(CSENS) by dividing the corresponding voltage readout by the value of R_26_ (see Figure 10).

(3) $$I_{PP+}=\frac{V_{p\_curt}}{R_{26}}I_{PP-}=\frac{V_{n\_curt}}{R_{26}},$$

By reversing the sampling (PWM1) and excitation (PWM0) phase, the obtained offset ADC voltages of entire signal-conditioning chain are subtracted from their in-phase readouts. The subtraction results in mutual cancellation of any offset DC voltages present in the signal chain. Conductivity at a measured temperature C(T), already expressed in (1), can be re-written using the quantities measured by the electronic module as follows:

(4) $$C\left(T\right)=\frac{k_{C}}{R_{C}}=k_{C}\cdot \frac{I_{PP+}-I_{PP-}}{V_{p_{volt}}-V_{n_{volt}}}$$

This result can be recalculated to a reference temperature (i.e., 25 °C) using (1) to obtain *C*_25_.

#### 2.5.2. Temperature Measurement Principle

Conductivity measuring system accuracy is strongly dependent on its temperature compensation, as described in Section 2.2; therefore, conductivity measurement devices have to implement effective and adjustable temperature compensation. Solution temperature coefficients are generally nonlinear and usually vary with the actual conductivity as well; therefore, the best calibration accuracy is achieved by measuring temperature in situ.

**Figure 17:**
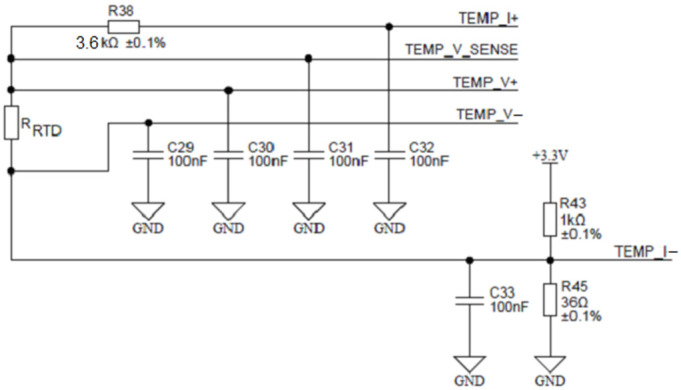
Configuration for four-wire RTD connection.

To achieve this, the ADuCM360 microcontroller contains two matched programmable excitation current sources which can be individually configured to provide a current output from 10 µA to 620 µA. In Figure 17, one such source is connected to the TEMP_I+ terminal and injects an excitation current of 100 µA into the series connection between the reference resistor R38 and the measured RTD resistor R_RTD_. The current returns through the TEMP_I- terminal. Either resistor voltage can be measured. The ability to inject current on the same pin, while being able to perform the ADC measurement, allows the ADuCM360 to easily perform two-wire, three-wire or four-wire RTD measurement configurations for either Pt100 or Pt1000 or it can be extended to any other RTD sensor type. Based on the ability of ADuCM360 to reconfigure/reconnect its programmable current sources, it is possible to implement automatic software detection of the RTD connection type, since mode switching is accomplished entirely by the software. Figure 17 shows the applied configuration for four-wire RTDs. The presented four-wire RTD measurement is fully ratiometric—the RRTD is depending solely on the temperature stability of the resistor R38. In addition, a four-wire configuration eliminates the error associated with the lead resistances. The presented case uses a buffered differential ADC configuration mode, which requires ADC input voltages greater than 100 mV. The latter is provided by the R_43_/R_45_ resistor divider that introduces 115 mV of bias voltage to the ADC input. As mentioned above, the ratio between the temperature resistor *R_RTD_* value and the reference resistance value *R*_38_ can be determined by measuring the ratio of voltages on the corresponding resistors:

(5) $$\frac{R_{RTD}}{R_{38}}=\frac{V_{RRTD}}{V_{R38}}$$

In order to cover the entire desired temperature measurement range (see Table 1), *R*_38_ must represent the maximal *R_RTD_* value on the highest measured temperature; in the presented case, *R*_38_ = 3.6 kΩ.

#### 2.5.3. Depth/Pressure Sensor Measurement Principle

**Figure 18:**
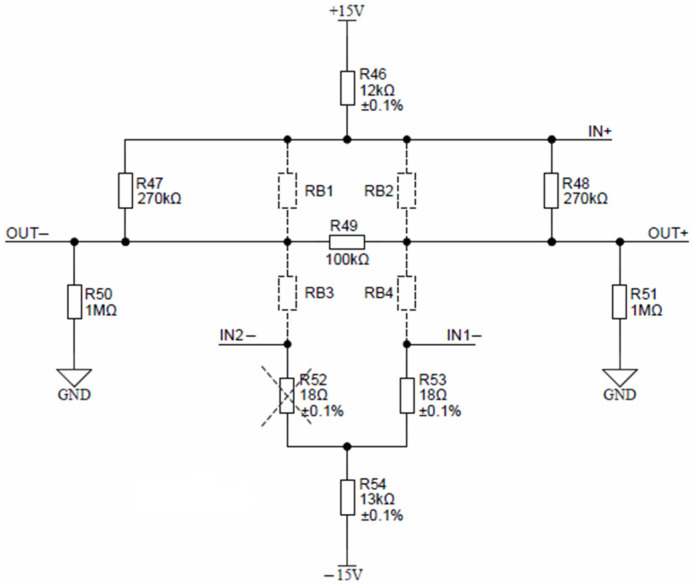
Keller pressure sensor connection detail.

The Keller piezoresistive pressure sensor, depicted in Figure 18, is comprised of resistors R_B1_, R_B2_, R_B3_ and R_B4_.

Nominal conditions require a constant current excitation of 1 mA, set by resistors R_46_ and R_54_. Pressure sensor offset can be reduced by selecting proper offset compensation resistors. Depending on the offset sign, either R_52_ or R_53_ is mounted. Sensor response can be linearized by mounting either the R_47_ or R_48_ resistor. If the sensor is absent, resistors R_49_, R_50_ and R_51_ provide zero output-voltage readout. According to the Keller sensor calibration table, the expected FS output voltage is around 200 mV at a maximal pressure; therefore, no external circuitry is needed for signal conditioning. Sensor output connections OUT− and OUT+ can be directly connected to differential inputs AIN10 and AIN11 of ADuCM360. Auto-ranging is achieved through the programmable gain amplifier located inside ADuCM360. The maximal 24-bit differential ADC measurement range is 1.2 V, which can then be further narrowed by selecting a gain of 2, 4, 8, 16, 32, 64 and 128. Each measurement range can be measured using the 24-bit ADC inside ADuCM360, resulting in the measurement of pressure sensor voltages down to a microvolt range. Pressure sensor signal conditioning was added onto the AIN10 and AIN11 differential inputs of ADuCM360. This microcontroller contains separate ADC0 and ADC1 units. The ADC1 interrupt handler (on_adc1) was changed to handle pressure and temperature sensors by alternating the ADC1 unit configuration. Furthermore, the software enables the setting of up to five pressure sensor calibration points. Typically, three points are provided by Keller with each pressure sensor. Pressure sensor evaluation is then performed using piecewise linear interpolation between the entered calibration points.

## 3. Measurement Setup

Two setups for the characterization and testing of CT sensors were applied. The first one is a setup for characterization using professional laboratory instruments (LCR meter HP4284A for characterization of conductivity sensor and Keithley KE2700 for temperature sensor) and the second one uses the electronics presented in this study. Both setups share the same HAAKE K20/DC3 temperature thermostated bath.

**Figure 19:**
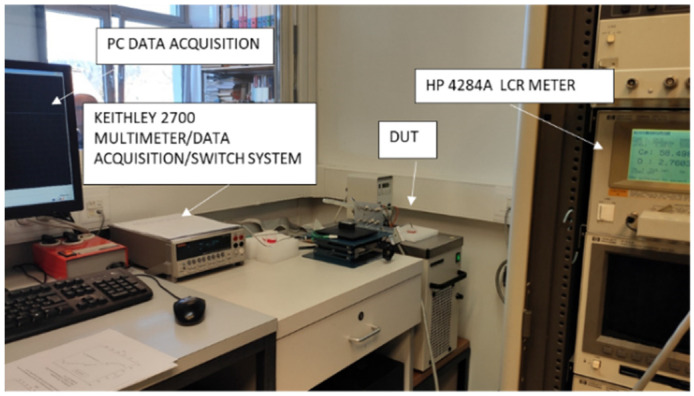
Measurement setup for CT sensor characterization by lab instruments. DUT is CT sensor inserted into Eppendorf tube and dipped into thermostated bath.

Laboratory tests were performed in an air-conditioned room at a temperature of 25 ± 1 °C and the device under test (DUT) was inserted into a HAAKE K20/DC3 temperature thermostated bath with a temperature accuracy of 0.01 °C. Before any measurement was performed, a one-hour temperature stabilization period of the test instruments was allowed, if not stated otherwise. Each setup, shown in Figure 19, also includes proper wiring and connections to connect the sensors with the instruments, and a container with a measured medium, which allows for repeated handling and thus avoids systematic errors.

## 4. Measurement Results

### 4.1. Characterization of Conductivity Sensor

The conductivity sensor transimpedance *Z* as a function of frequency has a frequency-independent plateau with a certain bandwidth determined by the cut-off frequencies, *f_low_* and *f_high_*. This response could be well modeled by an equivalent circuit of resistors and capacitors, as presented in the literature [7]. If the impedance *Z* at the plateau (phase angle *Θ* ≈ 0°) is given by *R_C_*, sensor conductivity *C*(*T*) can be expressed using (1). Below 10 kHz, the double-layer capacitance at the drive electrodes began to influence the impedance, while above 100 kHz, the parasitic capacitance of the conductivity cell substrate contaminated the conductivity measurements. It is therefore the range between 10 and 100 kHz where the measured impedance is dominated by seawater conductivity. In our case, an operating frequency of 15 kHz was selected for the device, as it is still high enough to minimize electrode double-layer serial resistance and can assure low noise level. In addition, a sufficiently low drive current was chosen to minimize the polarization effect of electrodes and, consequentially, their electro-corrosion. Electro-corrosion takes place predominantly when the drive electrodes are subjected to an AC signal with a DC offset or due to a high excitation current density, and can cause metal degradation that can lead to output drift during long-term measurements. Therefore, the measured signal should be as small as possible, but still high enough to obtain reliable measured results. For transimpedance measurements using an HP4284A LCR meter, the sinewave excitation voltage should be between 50 mV_RMS_ and 200 mV_RMS_. This limits the current in the drive electrode to below 0.5 mA, which corresponds to the current density of 0.25 mA/cm^2^, considering the electrode area of the chosen sensor. The measured signal of the voltage electrodes is consequentially at least in the range of a few mV. In all HP4284A measurements, if not stated otherwise, a 100 mV excitation voltage was applied. The same current density was achieved on the developed CTD electronics using a square wave excitation voltage *V_EXC_* of 1.6 V.

**Figure 20:**
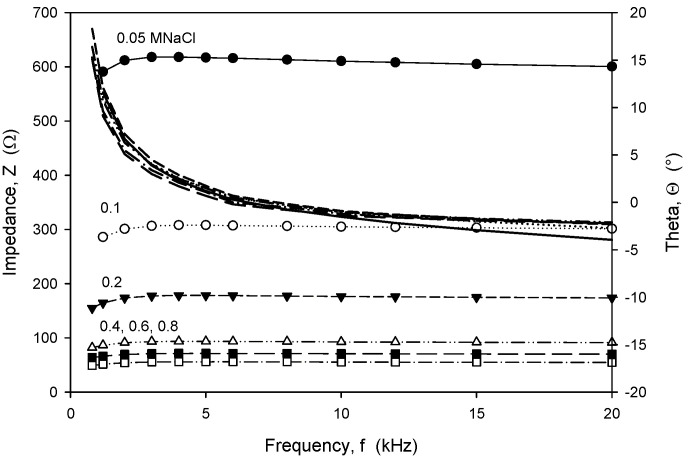
Frequency dependence of measured transimpedance and phase angle for six different concentrations of NaCl water solution. Measurement conditions: 25 °C, LCR meter, 100 mV and 8 kHz.

Frequency-dependent (trans)impedance at six concentrations of NaCl solution in the range from 0.05 M to 0.8 M was measured using an HP4284A LCR meter. The measured transimpedance (Z) and phase angle (*Θ*), depicted in Figure 20, show that in the frequency range from 8 kHz to 20 kHz, the character of impedance is almost ohmic, with a resulting small *Θ* of a few degrees, except for a NaCl concentration of 0.05 M. Afterwards, slightly higher frequency dependencies were obtained and a transit from inductive to capacitive impedance character.

**Figure 21:**
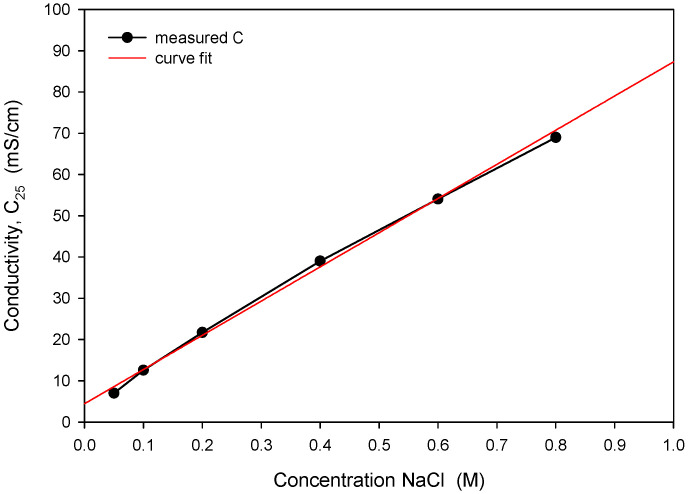
Measured conductivity dependence on NaCl concentration and determination of linear response.

**Figure 22:**
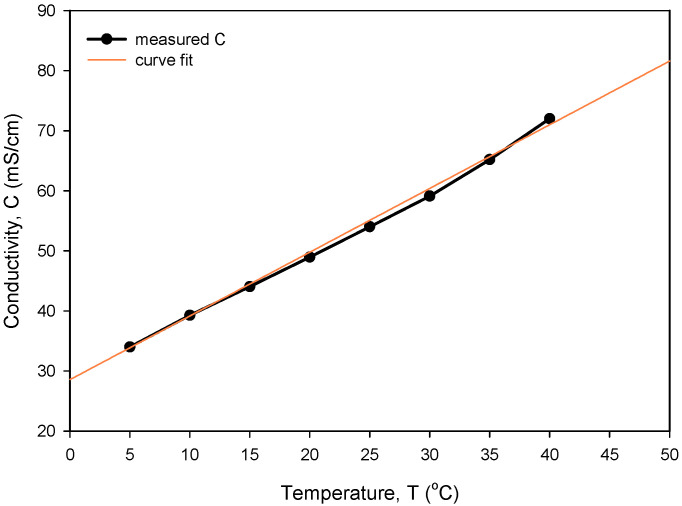
Temperature dependency of measured conductivity and determination of TC_C_.

The obtained optimal excitation frequency (i.e., 15 kHz) was then used in the measurement of conductivity vs. NaCl concentration (Figure 21) to determine sensor sensitivity and linearity. Conductivity of the NaCl solution increases linearly with concentration in intervals for typical seawater salinity. As depicted in Figure 21, measured conductivity is a linear function of NaCl concentration in the measured range from 0.05 to 0.8 M (NaCl), with a coefficient of determination *R_sq_* = 0.995. The conductivity cell constant (*k_C_*) is a geometrical parameter of electrodes related to the measured transimpedance as described in (1). By measuring the transimpedance *Z* at a known conductivity of the sample, *k_C_* can be determined. As shown in Table 2, the constant *k_C_* was determined for three different designs of the conductivity sensor. In addition, we have also made a variation in electrode design, where we changed the distance between the current and voltage electrode from 100 µm to 200 µm. We found that higher electrode spacing lowers the constant *k_C_* by around 10% and, consequently, the sensitivity of the conductivity sensor. As already noted, the conductivity of saline is a function of temperature. In Figure 22, we have determined the temperature coefficient of conductivity (*TC_C_*) for the 0.6 M NaCl solution by measuring the transimpedance (*Z*) vs. temperature (5 to 40 °C). The translation between *Z* and *C* was determined with (6), where *C* = 53.4 mS/cm at 0.6M NaCl @25 °C [24]. The temperature coefficient of conductivity (*TC_C_*) of 2.0%/°C was determined with the coefficient of determination *R_sq_* = 0.994, as shown in Figure 22.

The obtained measurements exhibit a slightly nonlinear dependency on concentration at the upper limit of the measurement range (around 0.8 M and 30 °C). Temperature compensation and characteristic linearization can be the subject of further improvements in the recalculation by using segmentation of the conductivity sensor evaluation (3) with coefficients obtained from a linear fitting model.

**Figure 23:**
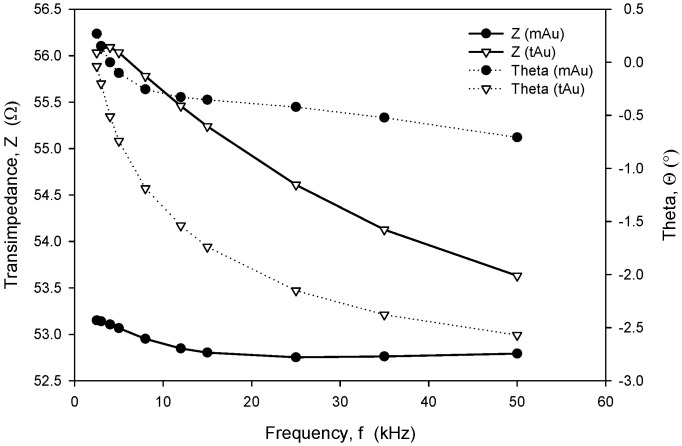
Frequency-dependent transimpedance and phase angle of microstructured and thin-film Au electrode. Measurement conditions: 25 °C (stabilized), 100 mV, 8 kHz and 0.6 M NaCl.

**Figure 24:**
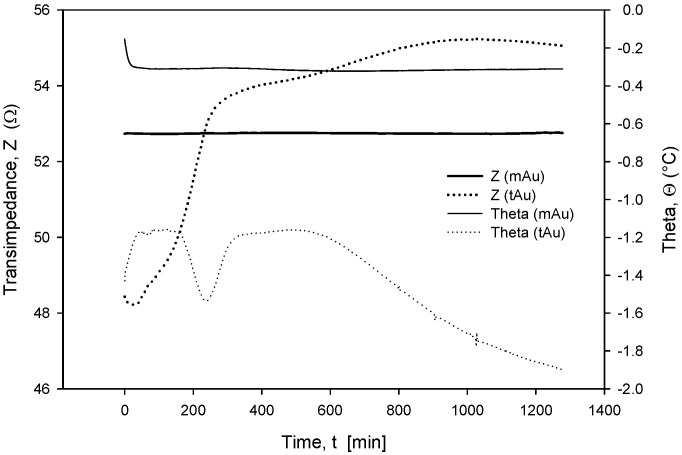
Time-dependent transimpedance and phase angle of microstructured and thin-film Au electrode. Measurement conditions: 25 °C (stabilized), 100 mV, 8 kHz and 0.6 M NaCl.

Long-term stability is one of the crucial parameters of conductivity sensors. While the accuracy is primarily related to the stability of the electronics, which should, among other things, perform temperature compensation of media, the main stability-related parameters are as follows: inertness of the electrode upon degradation (corrosion), biofouling, and the type of surface passivation and encapsulation of the chip in the sensor housing. Our investigation has also shown that the long-term stability of conductivity sensors is improved if thin-film Au electrode surfaces are modified through microstructuring, as described in Section 2.4. As shown in Figure 23 and Figure 24, microstructured Au electrodes (mAu in Figure 23 and Figure 24) exhibit a better frequency and time response in terms of faster stabilization of the measurement result compared to thin-film Au electrodes (tAu in Figure 23 and Figure 24). In addition, they also exhibit superior and long-term temporal stability with respect to thin-film Au electrodes.

**Figure 25:**
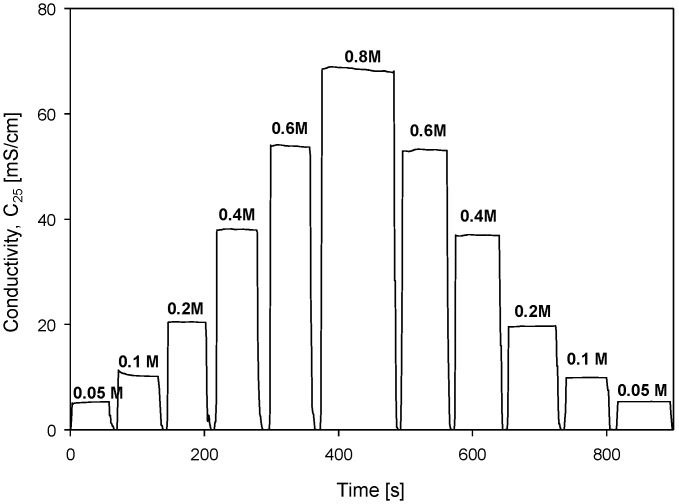
Conductivity measurements as function of stepwise increasing and decreasing of solution concentration. Parameter settings: 100 mV, f = 8 kHz, k_C_ = 2.80 cm^−1^ and TC_C_ = 2.0%/°C.

Conductivity cell hysteresis was investigated with stepwise changes in NaCl molarity, as shown in Figure 25.

Increasing steps of concentration (from 0.05 M to 0.8 M) were followed by a decreasing step (from 0.8 M to 0.05 M) direction. Each step was monitored for 1 min at each concentration and the result is presented in Figure 25. This somehow mimics the seawater currents or depth variations in salinity during probe dives. This information is relevant to evaluate electrode behavior in the scope of memory effect or transient charging in the vicinity of electrodes due to changes in ion concentrations.

### 4.2. Characterization of Thin-Film Ti RTD

Ti RTDs were characterized with a multimeter/data acquisition system Keithley 2700, which enables RTD measurements with an accuracy less than 0.03 Ω. For the tested Ti RTDs, the resistance accuracy to temperature accuracy was 2 m°C. A four-terminal connection configuration to the sensor was applied. The DC bias level should be high enough to overcome problems with the electronic noise of the system and adequately low to prevent self-heating of the sensor. Typically, a DC current of 100 µA is adequate for our RTD with a resistance of around 3000 Ω. In this case, the voltage of the temperature sensor is in the range of around a few hundred millivolts and the power dissipation ranges across tens of microwatts. The reference temperature resistance *R*_0_ was determined by measuring the CT sensor inserted into a Dewar flask in stabilized ice-cold water at an accuracy of 0.05 °C.

**Figure 26:**
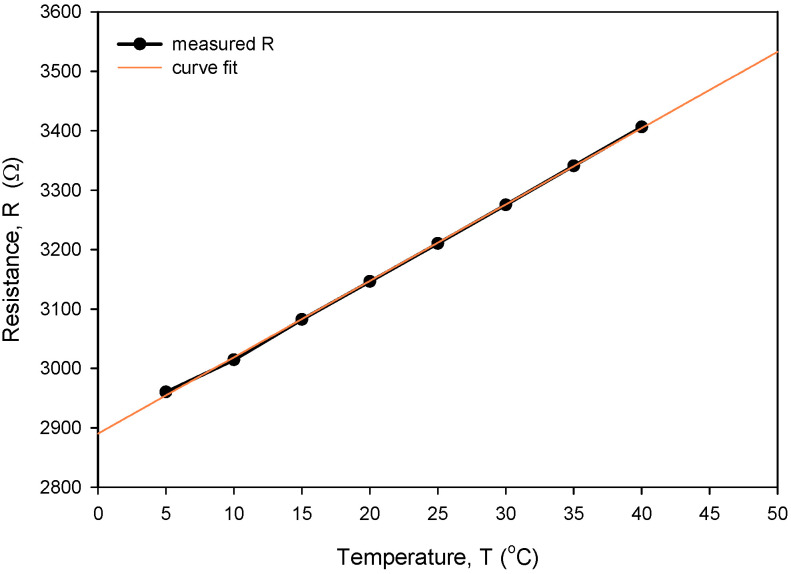
Determination of TC_R_ for our typical thin-film Ti RTD.

Figure 26 shows the result of the measurement resistance of a typical RTD that is independent of temperature in the range from 5 to 40 °C. The connected CT chip was inserted into an Eppendorf tube with a 0.6 M NaCl/water solution and dipped into a temperature-controlled Haake bath with a starting temperature of 5 °C. Resistance was measured using KE2700 in a four-wire configuration. Temperature was stepwise increased up to 40 °C in step increments of 5 °C, allowing for one-hour stabilization at each temperature. Temperature coefficients of resistivity for the thin-film Ti RTD (TCR) and extrapolated *R*_0_ at 0 °C were calculated using linear regression. Five resistance measurements were averaged to obtain a stable reading at each temperature.

**Figure 27:**
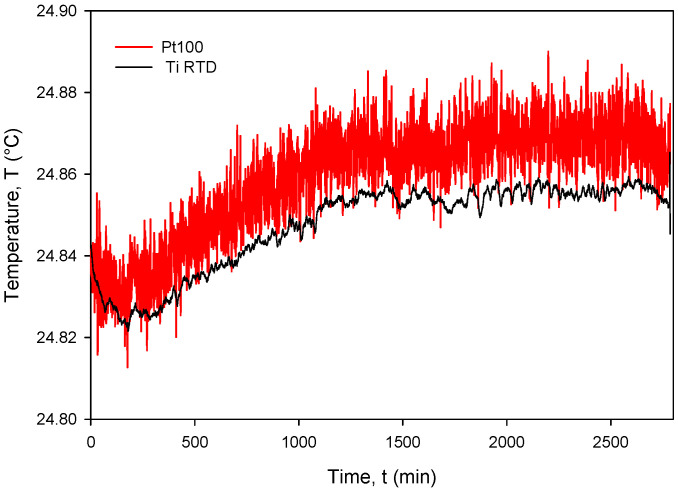
Long-term measurement and corresponding difference between the Ti RTD and PT100 temperature sensors.

**Figure 28:**
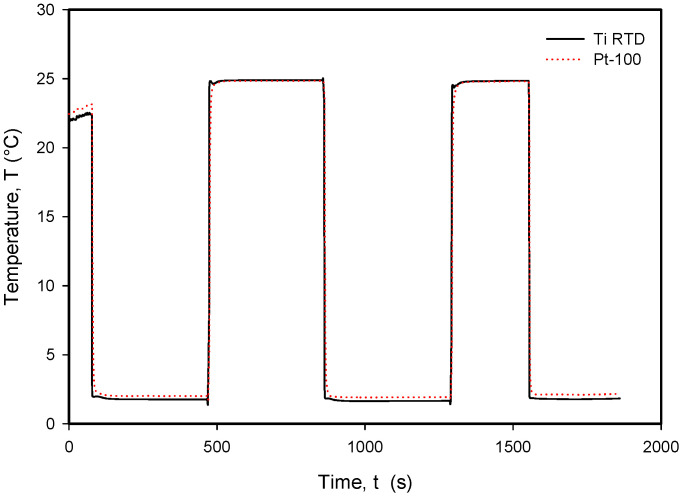
Long-term response to rapid temperature changes in compared Ti RTD and Pt100 temperature sensors.

**Figure 29:**
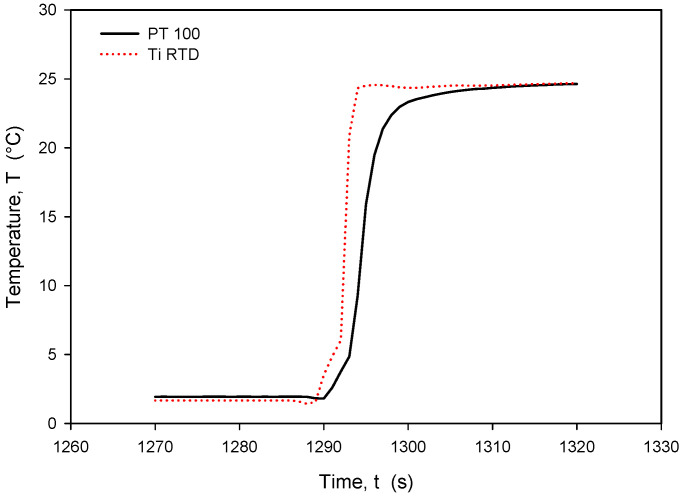
Time response of Ti RTD and PT100 during rapid temperature increase.

**Figure 30:**
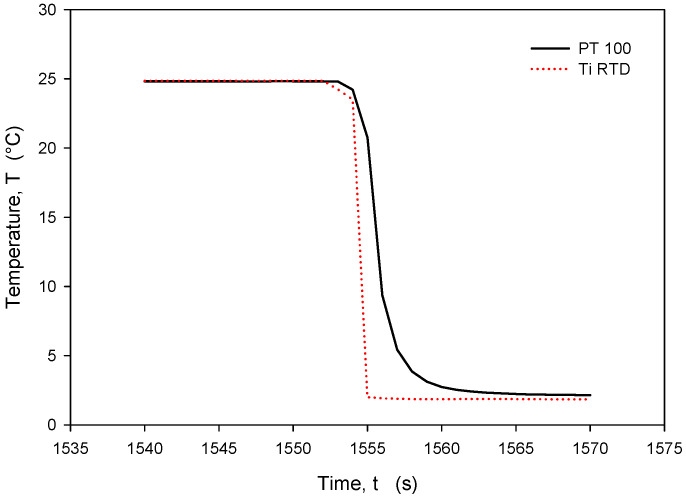
Time response of Ti RTD and Pt100 during rapid temperature decrease.

The calculated *R_sq_* value of 0.9998 indicates a good linear agreement between the measured (resistance) and predicted variables (temperature). The resistivity temperature coefficient (*TC_R_*) of 4450 ppm/°C at T = 0 °C was determined based on the good agreement between the measurement and numerical fit (*R_sq_* = 0.9998). Calibration was performed by comparing temperature measurements of the Ti RTD with reference Pt100 (class A) readouts. Figure 27, Figure 28, Figure 29 and Figure 30 show the long-term measurement and corresponding difference between the two compared RTDs. Temperature measurement error can be estimated as the difference between readouts from Figure 27.

The estimated long-term stability of less than 0.025 °C (see Figure 27) over 2 days was obtained on different occasions and testing different sensors. This implies that Ti RTD accuracy is in the same range as the reference sensor class A. Moreover, the thin-film Ti RTD also exhibits much lower measured noise compared to the Pt100 temperature sensor built in our custom-designed metal housing (metal tube) due to the higher value of serial resistance of theh wires and the KE2700 switching matrix relays.

It should be noted that the resistance of an individual thin-film Ti resistor may vary due to fabrication process tolerance; therefore, it is mandatory to determine *TC_R_* and *R*_0_ values for each individual sensor batch. The time response of Pt100 built in the metal tube and firmly positioned 5 mm next to the RTD was measured and is presented in Figure 28. In this experiment, the Ti RTD sensor was encapsulated in housing, depicted in Figure 5b. The measured noise for the Pt100 sensor is ±15 m°C, while the presented Ti RTD exhibits noise of ±3 m°C. The setup allowed for the physical transfer of the sensors between the Haake bath at 25° and the 100 mL Eppendorf tube filled with DI water and a thermoflask with ice-cooled DI water at 2.2 °C. Several transfers between the two media were executed, performed with good repeatability. Figure 29 and Figure 30 show the transition rise and fall time details from Figure 28 for the Pt100 and Ti RTD sensors.

A comparison of the temperature transition times shows that Pt100 is slower in reaching 90% of the final value. The thin-film Ti RTD shows a slightly lower time constant, i.e., a faster response.

Table 3 shows the comparison between the 10% and 90% rise- and fall-time slope for the PT100 sensor and the Ti RTD sensor in the linear part of the temperature transition.

### 4.3. Characterization of CTD Sensors with Developed Electronics

The developed electronics, as shown in Section 2.5., considered all requirements and parameters needed in developed CT sensors. The data logging software was designed to enable graphical representation of continuously and simultaneously acquired CTD parameters. The most important data are calculated conductivity, temperature and depth, while excitation parameters such as conductance voltages (*V_PP+_* and *V_PP−_*) and currents (*I_PP+_* and *I_PP−_*) are also monitored to provide a more detailed insight into the operation of the electronics and sensor.

**Figure 31:**
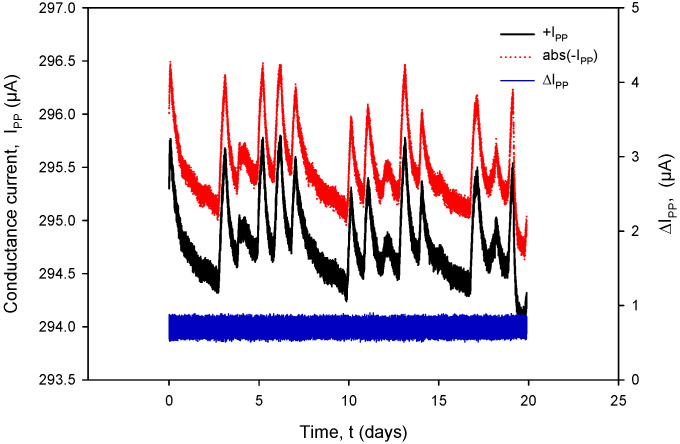
Graphical representation of continuously acquired instrument parameters using custom data logging software. Applied test conditions: V_EXC_ = 1.6 V, f = 15 kHz and k_C_ = 1 cm^−1^.

**Figure 32:**
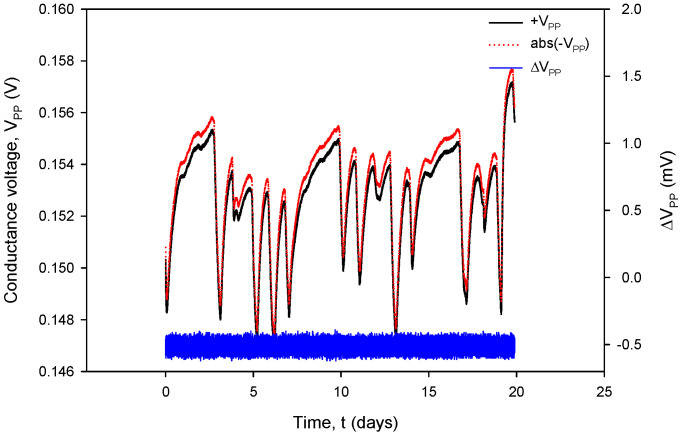
Graphical representation of continuously acquired instrument parameters using custom data logging software. Applied test conditions: V_EXC_ = 1.6 V, f = 15 kHz and k_C_ = 1.

Additional acquisition results, used for the calculation of *R_C_* in (1), are shown in Figure 31 and Figure 32. Conductivity sensor excitation was configured with *V_EXC_* = 1.6 V, *f_EXC_* = 15 kHz, *k_C_* = 1 cm^−1^ and *TC_C_* = 2.0%/°C. Measurements were taken over 20 days in room-temperature conditions in a 0.06 M NaCl solution at a rate of two samples per minute. The conductivity result was multiplied by the real value of *k_C_*, which is 2.80 cm^−1^ for the conductivity sensor of type C1 (Table 2). During the 20 days of continuous measurement, the room temperature varied between 21 °C and 24 °C.

Figure 31 shows the conductivity sensor currents: a positive measurement cycle value of *I_PP+_* and an absolute value during the negative measurement cycle of *I_PP−_* and their difference (blue). During normal conductivity sensor operation (e.g., electrode corrosion), the current difference value remains within 0.4% FS.

Figure 32, coherent with the time scale in Figure 31, shows the absolute value of conductivity sensor voltage during the negative measurement cycle (*V_PP−_*), the voltage measurement during the positive cycle (*V_PP+_*) and their difference (blue). Again, during normal sensor operation, the voltage difference value remains within 0.4% FS.

**Figure 33:**
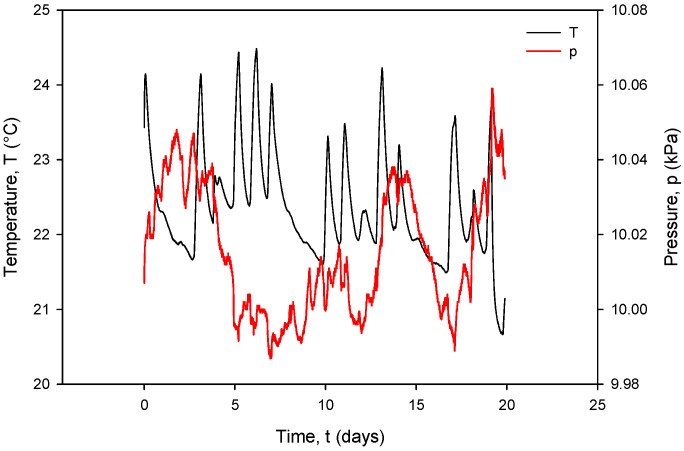
Ti RTD temperature (black) and pressure (red) under room-temperature conditions.

Figure 33, coherent with the time scale in Figure 31, shows the Ti RTD temperature *T*(°C) (black) and pressure *P*(kPa) (red) under room-temperature conditions. The observed temperature variations between 20.5 °C and 24 °C coincide with the conductivity cell current and voltage variations.

**Figure 34:**
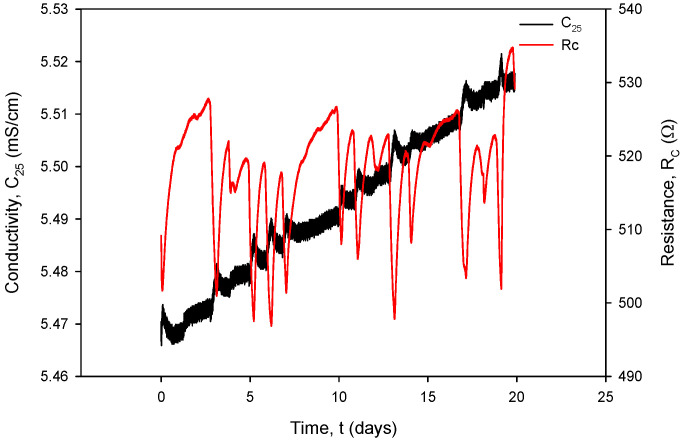
Temperature-compensated conductivity value (black) and resistance value (red).

Figure 34 shows the temperature-compensated conductivity value *σ* (S/cm) (black) and resistance value *R_C_* (red), calculated using (1). Typical measurement results from Figure 31, Figure 32, Figure 33 and Figure 34 are *I_PP+_* = 295 µA, *V_PP+_* = 150 mV, *R*_COND_ = 500 Ω, *T* = 23 °C, *C* = 2.2 mS/cm and atmospheric pressure. 

It can also be seen that time variation in the conductance currents |*I_PP_*| (Figure 31) and voltages |*V_PP_*| (Figure 32) as well as the resistance *R_C_* (Figure 34) exactly follow the temperature *T* of the conductivity cell, measured by the on-chip Ti RTD temperature sensor (Figure 33).

The variations in |*I_PP_*| and |*V_PP_*| change with increasing NaCl concentration: if the concentration was increased from 0.06 M to 0.6 M, typical |*I_PP_*| readings would increase from 295 µA to 318 µA, while typical |*V_PP_*| readouts would decrease from 150 mV to 16.7 mV. In the case of 0.6 M, current and voltage variations are even smaller: |*I_PP_*| variation is 0.693 µA and |*V_PP_*| variation is 52.81 µV; therefore, we can safely apply a variation of 0.4% as the worst (0.06 M) case of the two.

Figure 34 shows the effectiveness of temperature compensation of conductivity using the compensation formula (Formula (2)) in the case of the 0.06 M solution. If the conductivity temperature compensation is disabled, a variation of 3 °C in analyte temperature results in a resistance variation of Δ*R_C_* = 40 Ω, which translates to a change in conductivity of ΔC(T) = 151 µS/cm (*k_C_* = 1 cm^−1^). 

**Figure 35:**
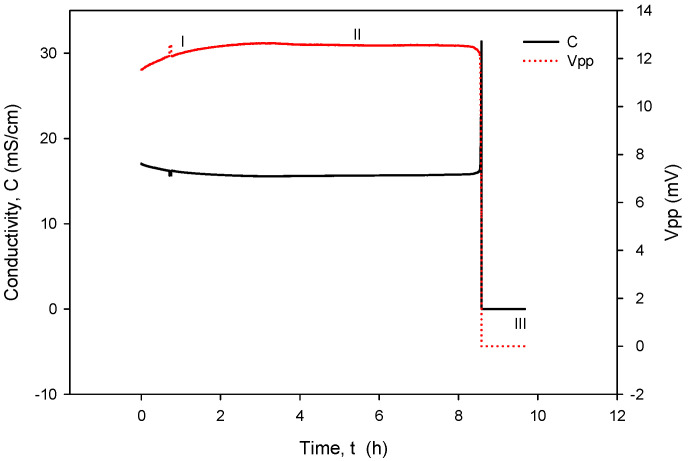
Detection mechanism for electrode corrosion process.

When operational, conductivity readouts are limited only by sensor signal-conditioning noise. Conductivity measurement noise can be estimated to ±1 µS/cm. The long-term time drift of the measured conductivity of 0.1%/day can be attributed to water evaporation from the measured sample, e.g., a poorly sealed Eppendorf vial, while the spikes in measurements are a consequence of calculations due to rapid temperature changes from decreasing to increasing. Figure 35 depicts a phenomenon which occurs if coupling resistors *R*_21_ and *R*_22_ (Figure 13) are omitted. If the resistors are included and the conditions are constant (T, medium), then the electrode voltage difference *V_PP_* is within the abovementioned variations and the conductivity remains time-constant (Figure 31, Figure 32, Figure 33 and Figure 34). On the other hand, when coupling resistors *R*_20_ and *R*_21_ are omitted (as in Figure 35), different variations in conductivity cell voltages *|V_PP_|* represent means for erroneous conductivity sensor readout detection. In Figure 35, if the conditions are constant (T, medium) and the |*V_PP+_*| and |*V_PP−_*| both increase with time, this indicates that the electrode polarization potential (region I) is building up. After some time, it reaches a steady state (region II) where electrode corrosion takes place, followed by a sudden voltage drop and erratic voltage response (region III), where the electrode is interrupted (depicted in Figure 12). Such an abrupt change in voltage difference also affects the conductivity result, as shown in Figure 35.

The voltage electrode response in region I demonstrates the internal charging of voltage electrodes, i.e., an increase in the electrical potential between the voltage and current electrodes, which takes about 2 to 3 h, as shown in Figure 35. In region II, the electrochemical redox potential becomes large enough to transport Au from the voltage electrode to the adjacent current electrode (corrosion electrode). Finally (region III), a constant potential is maintained and after 8 to 10 h, discontinuity of the Au electrode occurs. The voltage electrode failure point usually occurs at the boundary of the passivated layer (Figure 12).

**Figure 36:**
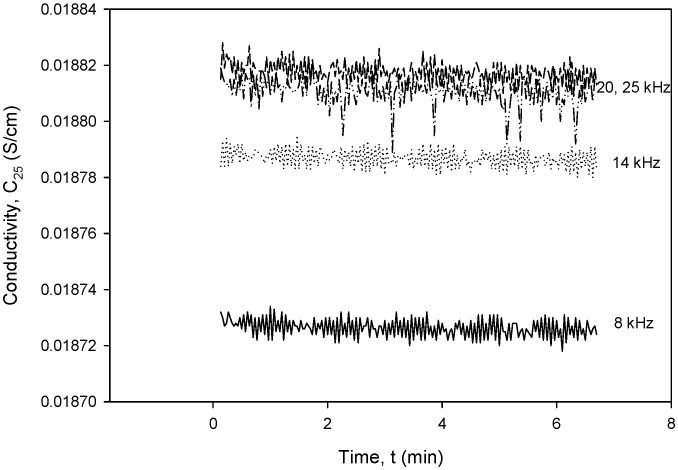
Conductivity cell noise evaluation for excitation frequency range between 8 and 20 kHz.

**Figure 37:**
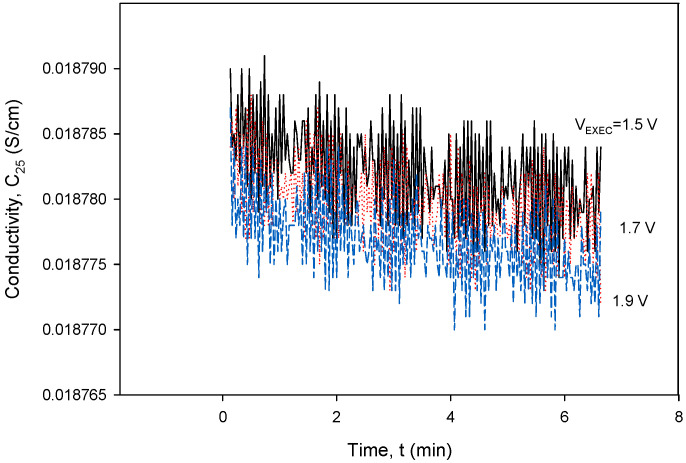
Conductivity cell noise evaluation for excitation voltage range between 1.5 and 1.9 V at 8 kHz. Parameter settings: *k_C_* = 1 cm^−1^ and *TC_C_* = 2.0%/°C.

To obtain the optimal operating parameters for the presented conductivity cell, separate frequency (Figure 36) and excitation voltage (Figure 37) scans were performed using the designed CTD electronics. Figure 36 and Figure 37 show the result of the dependency of excitation frequency and voltage variation on conductivity measurements.

Figure 36 shows the dependency of conductivity on excitation frequency in the range from 8 to 20 kHz for a constant excitation voltage of 1.6 V. It can be seen that until a frequency of 20 kHz, the noise is constant at ±3.5 µS/cm and starts to increase above it, so that at 25 kHz, it almost doubles. The measured noise at 8 kHz is within ±10 µS/cm.

Figure 37 shows a relatively small impact of *V_EXC_* variation on conductivity in the investigated voltage range. In order to reduce the sensor’s power consumption, the excitation current (*I_PP_*) should be kept as low as possible, but still high enough to be able to measure the concentration of the NaCl solution (70 mS) to be *V_PP_* > 10 mV. As can be seen from both Figure 36 and Figure 37, even a substantial change in excitation frequency and/or voltage does not cause any significant change in the conductivity result, since a relative change in frequency (Δ*f*/*f*) or excitation voltage (Δ*V_EXC_*/*V_EXC_*) of 10% results in a conductivity measurement error of 0.1%.

## 5. Conclusions

The design and characterization of a cost-efficient oceanographic instrument for the measurement of conductivity, temperature and depth of seawater was presented. The developed instrument enables the measurement of seawater conductivity in range from 4 mS/cm to 70 mS/cm. Conductivity measurement is temperature-compensated in the range from 2 °C to 40 °C. The depth/pressure measurement range is up to 2000 m. Temperature and conductivity sensor performance was assessed using laboratory equipment and the designed electronics. Conductivity sensor excitation parameters were analyzed with respect to noise level response and a set of optimal parameters was determined. The effectiveness of the proposed temperature compensation approach was measured. Dedicated analysis software was programmed and used to monitor the detailed conductivity sensor properties. A conductivity sensor readout validity criterion was developed from monitoring the variations between current and voltage electrode responses. A fixed variation of 0.4% between current and voltage electrode readouts was established as a criterion for a valid sensor response. The measurement range of the conductivity sensor could be further extended by segmenting the sensor characteristics, which will minimize the effect of sensor nonlinearity at higher conductivity values.

## Figures and Tables

**Table 1:** Technical characteristics of CTD instrument.

Parameter	Conductivity	Temperature	Pressure
Measurement range	4 to 70 mS/cm	0–40 °C	0…200 bar
Temperature range	0 °C to 40 °C	/	−20…85 °C
Temperature coefficient	2%/°C @ 0.6 M NaCl, 25 °C	4450 ppm/°C @ 0.6 M NaCl	/
Accuracy	±0.1 mS/cm	/	±0.5%FS
Resolution	0.01 mS/cm	±0.01 °C	0.1 bar

**Table 2:** Electrode design geometry of conductivity sensor and corresponding cell constant, *k_c_*.

Chip	l (cm)	d (cm)	2a (cm)	A = l × 2a (cm^2^)	kc (cm^−1^)
C1	0.25	0.65	0.08	0.02	2.80
C2	0.25	0.69	0.04	0.01	3.15
C3	0.125	0.65	0.08	0.01	3.35

**Table 3:** Comparison between rise- and fall-time gradients of PT100 and Ti RTD temperature sensors.

Sensor Type	Rise-Time Gradient (ms/°C)	Fall-Time Gradient (ms/°C)
Pt100	370	312
Ti RTD	117	87

## Data Availability

Data are contained within the article.
